# Early Graft Function in Deceased Donor Renal Recipients: Role of N-Acetylcysteine

**DOI:** 10.22037/ijpr.2019.15546.13167

**Published:** 2020

**Authors:** Atieh Modarresi, Mohsen Nafar, Zahra Sahraei, Jamshid Salamzadeh, Shadi Ziaie

**Affiliations:** a *Research Center for Rational Use of Drugs, Tehran University of Medical Sciences, Tehran, Iran. *; b *Department of Clinical Pharmacy, School of Pharmacy, Shahid Beheshti University of Medical Sciences, Tehran, Iran.*; c *Chronic Kidney Disease Research Center, Shahid Labbafinejad Medical Center, Shahid Beheshti University of Medical Sciences, Tehran, Iran.*; d *Loghman Hakim Hospital, Shahid Beheshti University of Medical Sciences, Tehran, Iran.*

**Keywords:** Transplants, Kidney Transplantation, Reperfusion Injury, Acetylcysteine, Lipocalin-2, Glomerular Filtration Rate

## Abstract

Reduced graft function (RGF) in donor renal transplant recipients is caused by oxidative damage due to extensive ischemia-reperfusion (I/R) injury during transplantation. Neutrophil gelatinase-associated lipocalin (NGAL) is a promising biomarker to detect tubular injury early after renal transplantation. N-acetylcysteine (NAC) is a potent antioxidant that can reduce I/R injury by improving oxidative damage. The aim of the present study is to assess the efficacy of NAC in improving graft function and reducing renal tubular injury in deceased donor renal transplant recipients. A double-blind, randomized clinical trial was conducted on 50 deceased donor renal transplant recipients. The patients were randomized into two groups, receiving either 600 mg NAC twice daily, or placebo (days 0 to 5). Results were assessed based on the rate of RGF, levels of plasma NGAL (p-NGAL) and the estimated glomerular filtration rate (eGFR). The rate of RGF was significantly lower in the patients receiving NAC vs. placebo (21.4% vs. 50%). The measurement of p-NGAL levels showed that the patients in the NAC group had significantly greater reduction of p-NGAL by both days 1 and 5 post-transplantation than those in the placebo group. A near steady-state eGFR level was reached by week 1 in the NAC group, however, the improvement of eGFR was significantly slower in the placebo group and a near steady-state was only achieved by week 4. NAC has promising potential in reducing tubular injury and improving graft function, evidenced by significant reduction in the rate of RGF and levels of p-NGAL.

## Introduction

Reduced graft function (RGF) defined as delayed graft function (DGF) or slow graft function (SGF) is a continuous spectrum of ischemia-reperfusion (I/R) related acute kidney injuries (AKI) that occur in more than 20% of kidney transplant recipients (1, 2). Delayed graft function, the most severe form of AKI, is highly detrimental to kidney transplant recipients as it increases the risk for both acute and chronic rejection as well as long-term graft loss (3). Several studies reported that not only the patients with DGF but also the patients with SGF showed worse short- and long-term outcomes than those with immediate graft function (IGF), with the increased incidence of acute rejection in the first 6 months and a negative impact on long-term graft and patient survival rates (4, 5). RGF is mostly caused by oxidative damage to the graft due to extensive I/R injury during transplantation. It is more prevalent in deceased than living donors because of the significant ischemic injury of the graft (6). I/R causes cell death, inflammatory response, immunologic activation, tissue fibrosis and impaired renal microcirculation (1, 7). Consequently, many pharmacologic strategies, especially antioxidant agents, have been proposed to limit oxidative stress induced by I/R injury (8, 9). N-acetylcysteine (NAC) is a potent antioxidant that regenerates glutathione stores and scavenges oxygen-free radicals (10, 11). Several studies have reported the efficacy of NAC in reducing the I/R injury and improving graft function in kidney transplantation (12, 13). In a study on stable renal transplant recipients, NAC improved both serum creatinine and estimated glomerular filtration rate (eGFR) (14). The effect of NAC in reducing oxidative stress markers has been demonstrated by several previous studies (12). Clinically, serum creatinine is the most widely used marker to assess graft function. However, serum creatinine is a nonspecific biomarker for I/R and has low sensitivity for monitoring early graft recovery following renal transplantation (15, 16). An early and more specific biomarker can be helpful in early diagnosis of AKI and assessing the risk of developing RGF (17, 18). Neutrophil gelatinase-associated lipocalin (NGAL) is a promising biomarker to detect tubular injury early after renal transplantation (19). Studies have shown that urine and plasma NGAL correlate with graft function post-transplantation and are early markers for AKI diagnosis (20-22). Given its proven antioxidant properties and vasodilatory effects, this study aimed to assess the efficacy of NAC in improving graft function in deceased donor renal transplant recipients. In addition, plasma NGAL (p-NGAL) was used to measure the effect of NAC in reducing renal tubular injury. The novelty of this study is to assess the efficacy of NAC in improving graft function and reducing renal tubular injury in deceased donor renal transplant recipients which is determined by creatinine reduction ratio (CRR) and NGAL.

## Experimental

*Study design*

A prospective, double-blind, randomized, placebo-controlled clinical trial was conducted on deceased donor renal transplant recipients in Shahid Labbafinejad Hospital in Tehran, Iran from May 2014 to December 2015. Taking into account the trial’s inclusion and exclusion criteria, the recruited patients were randomized to two groups, receiving either NAC or placebo in addition to the existing immunosuppressant protocol. Ten tablets of 600 mg NAC (ACC long Hexal, Germany) or placebo with identical appearance were administered: one tablet within 2 h before and 9 tablets, twice daily, during the five consecutive days after transplantation. The placebo also was an effervescent tablet which was mixture of sodium bicarbonate and citric acid. The study was done in accordance with the declaration of Helsinki. The trial was registered in the Iranian Registry of Clinical Trials (www.irct.ir, registration number: IRCT2014090214693N4). It was also approved by the ethics committee of Shahid Labbafinejad Hospital and the Urology and Nephrology Research Center, Tehran, Iran. Written informed consents were obtained from all the patients prior to the enrollment. The consent form described the study, outlined the possible risks and indicated that an experimental medication or placebo would be given twice daily for five days.

*Inclusion and exclusion criteria*

Adult recipients of heart-beating deceased donor renal transplant, aged between 18 to 75 years were assessed for medical history and the patients with any of the following criteria were excluded: 1) preemptive kidney transplantation; 2) Warm ischemia in which lasts for more than 20 min 3) second or multi-organ transplantation; 4) history of NAC consumption within one month before transplantation; 5) history of sensitivity to sulfa drugs; 6) history of coronary artery bypass grafting (CABG) or stroke within 6 months before transplantation; 7) panel reactive antibody (PRA) of >30%. Exclusions 3 and 7 were considered because of different induction immunosuppressive regimen being considered for such patients in the study center. In addition, warm ischemia was considered as time of cardiac arrest till cannulation and perfusion of aorta (23).

*Transplantation protocol of study center*

The patients receive methylprednisolone (200 mg), mycophenolate mofetil (2.0 g) and either of tacrolimus (0.1 mg/kg) or cyclosporine (7 mg/kg/day) preoperatively. Induction polyclonal antibody (thymoglobulin) is indicated in the patients with PRA >30%, history of previous transplantation or multi-organ transplant; however, as noted in the inclusion and exclusion section, the patients receiving thymoglobulin were excluded from the study to standardize the immunosuppressive regimen in the trial. Prednisolone (2 mg/kg, maximum dose 120 mg/day) is started on the first day post-transplantation and tapered to reach a dose of 5-7.5 mg/day in three months. Postoperative does of tacrolimus or cyclosporine is adjusted to achieve target trough level of 7-10 ng/mL and 150-300 ng/mL, respectively, during the first three months after transplantation. The Patients receive mycophenolate mofetil 500 mg or 1.0 g twice daily in combination with tacrolimus or cyclosporine, respectively. Thymoglobulin is administered to the patients who develop DGF.

*Data Collection*

At baseline, the following data were recorded: demographic information and clinical characteristics of recipients and donors, cold ischemia time, and recipients’ drug history and PRA. Following transplantation, the estimated glomerular filtration rate (eGFR) was calculated at weeks 1, 2, 4, 8, and 12 from measured serum creatinine levels. The calculation of eGFR was based on the Modification of Diet in Renal Disease (MDRD) equation. In addition, the occurrence of slow and delayed graft function, biopsy-proven acute rejection and graft loss were recorded. Fasting venous blood samples were taken from all the recipients at baseline (pre-transplant) and on the first and fifth days after transplantation. The blood samples were centrifuged at 3500 rpm at 4 °C for 10 minutes and plasma was dispensed into micro tubes and stored at -80 °C. The preoperative sample was taken within 2 h before transplantation (day 0) and the two post-transplant samples were taken 24 h following the surgery (day 1) and on the fifth day six h after the last dose of NAC/placebo consumption (day 5). Plasma NGAL levels were measured using a research-based enzyme-linked immunosorbent assay (ELISA) from BioPorto Diagnostics (Gentofte, Denmark). The measurement was carried out in accordance with the instructions given by the manufacturer.

*Outcomes*

The primary outcome was the comparison between the two study groups in the number of RGF, defined as a CRR between day 0 (pre-transplantation) and day 7 post-transplantation of ≤70% requiring dialysis (DGF) or without dialysis (SGF). IGF was defined as a CRR between day 0 and day 7 of ≥70% (5). 

The secondary outcome was the comparison between the two groups in p-NGAL levels measured on days 0, 1, and 5 and eGFR of weeks 1, 2, 4, 8 and 12. 

*Sample size, randomization and blinding*

Based on standardized difference (difference/SD) of 0.75, power of 80% and alpha = 0.05, a sample size of 56 (28 in each group) was calculated. The patients were randomized to receive either NAC or placebo using a simple computerized randomization program. The placebo tablets were made in the School of Pharmacy, Shahid Beheshti University of Medical Sciences with the same appearance and packaging to those of NAC. Staff responsible for preparation of trial medications, samples collection and randomization process were not further involved in the study. The initiation of post-transplant dialysis was decided independently by the physicians. The patients, physicians, nurses and statistician were blind to allocation.

*Statistical analysis*

IBM SPSS Statistic 21.0 (SPSS Inc., Chicago, IL, USA) was used for statistical analyses. *P* value of 0.05 was considered as significant. All interval variables were tested for normality of distribution using Kolmogorov–Smirnov test. T-test and Mann-Whitney’s U test were used for data with normal and non-normal distribution, respectively. Chi-square and Fisher’s exact tests were used for categorical data. Nominal variables were reported as number (%) and continuous variables as mean ± standard deviation (SD) or median (IQR; interquartile range) for normal and non-normal distributions, respectively. 

Mixed ANOVA, with time as the within-subjects factor and group (NAC vs. placebo) as the between-subjects factor, was used for the analysis of variables measured at different time points, including p-NGAL and eGFR. The homogeneity of variance was verified using Levene’s test. Results of Greenhouse-Geisser correction were reported whenever Mauchly’s sphericity test was significant. When the interaction between time × group was significant, post-hoc analysis based on pairwise comparisons was conducted. P value less than 0.05 also was considered as significance level.

## Results

*Baseline characteristics*

**Figure 1:**
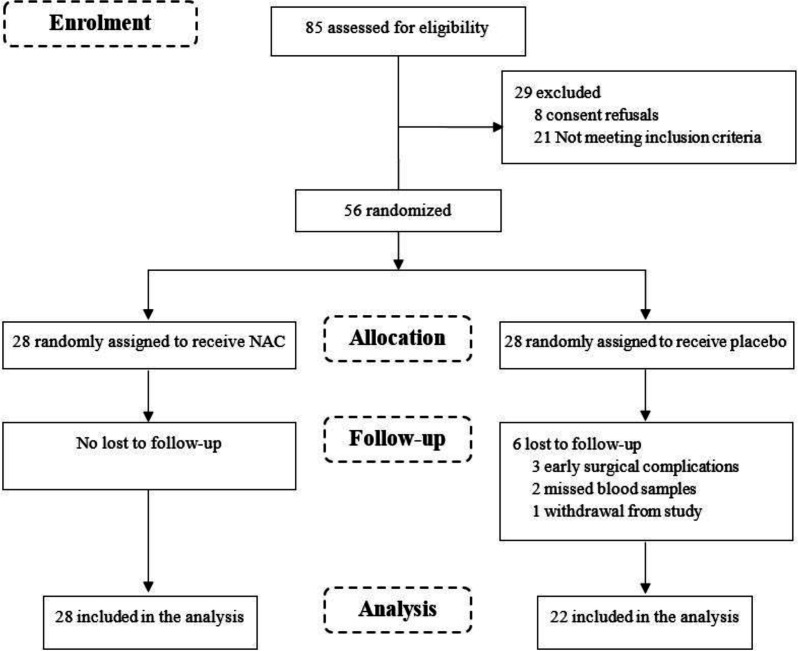
Randomization, treatment and follow-up procedures

A total of 85 deceased donor renal transplant recipients were screened for eligibility, of whom 56 patients, were randomized into two groups of 28, met the inclusion criteria and consented to participate in the study (Figure 1). 

During the course of the trial, the following exclusions, all in the placebo group, were made: 3 patients had early surgical complication, 2 patients had missed blood samples and one patient withdrew from the study. Consequently, 50 patients including 28 in the NAC group and 22 in the placebo group were considered in the analysis. The results from per-protocol analysis showed that the patients were aged between 18-64 years (mean 43.5 ± 12.2) and 70% male. The mean duration of dialysis was 1.3 ± 1.1 years and all the patients had negative PRA. The baseline characteristics and medication history of donors and recipients and the cold ischemia time were not statistically different between the NAC and placebo groups (Table 1).

*Early graft function*

**Figure 2:**
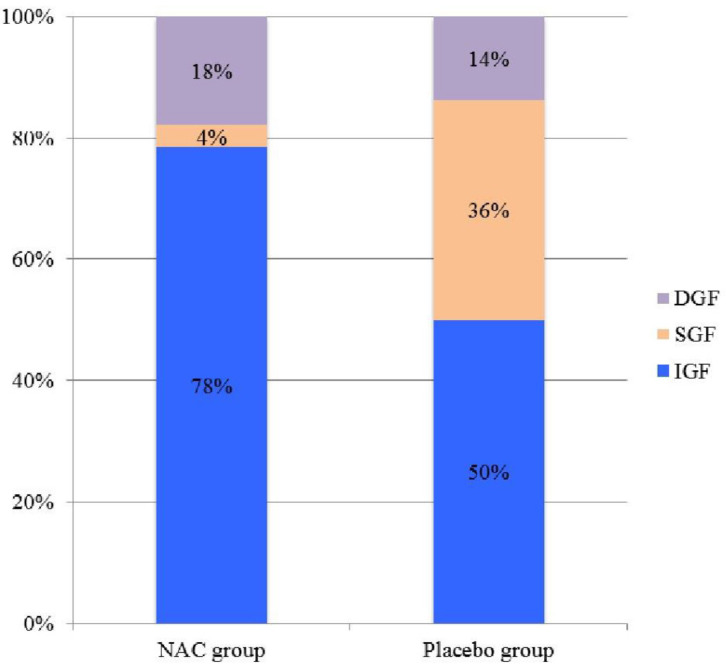
Early graft function. IGF, immediate graft function; SGF, slow graft function; DGF, delayed graft function

Of the 50 recipients of deceased renal transplants in the study, IGF was observed in 33 (66%), SGF in 9 (18%) and DGF in 8 (16%) patients (Table 2, Figure 2).

The number of RGF (DGF + SGF) was 6 (21.4%) vs. 11 (50%) and the number of IGF was 22 (78.6%) vs. 11 (50%) in the NAC and placebo groups, respectively (*p = *0.03). The length of dialysis in DGF patients was 12 days (6-18) vs. 14 days (9-24) in the NAC and placebo groups, respectively (*p* = 0.15).

*p-NGAL level*

**Figure 3:**
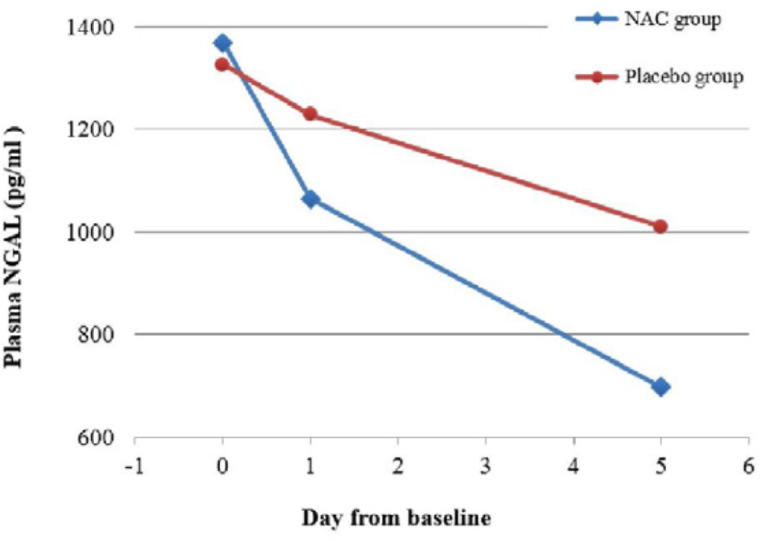
Plasma NGAL level during study intervention

There was significant difference across the three time points (days 0, 1 and 5) and between the two study groups in p-NGAL (Table 2). Most importantly, there was a significant interaction between time and group, indicating that the change of p-NGAL between the two groups over the three time points were significantly different (*p = *0.003). The post-hoc analysis showed that there was no significant difference in the p-NGAL between groups at baseline (*p* = 0.36), but the difference became significant at day 1 (*p *= 0.008) and day 5 (*p *= 0.004). The p-NGAL reduction in the NAC and placebo groups was respectively 22.2 % vs. 7.3 % by day 1, and 49.1 % vs. 23.8 % by day 5. Considering the groups separately, the p-NGAL in the NAC group reduced significantly from baseline to day 1 (*p* < 0.001) and from day 1 to day 5 (*p* < 0.001). However, the change of p-NGAL in the placebo group from baseline to day 1 was nonsignificant (*p* = 0.16), followed by a significant reduction from day 1 to day 5 (*p* = 0.009). Figure 3 shows the change in p-NGAL for both groups across the three time points.

*eGFR*

**Figure 4.**
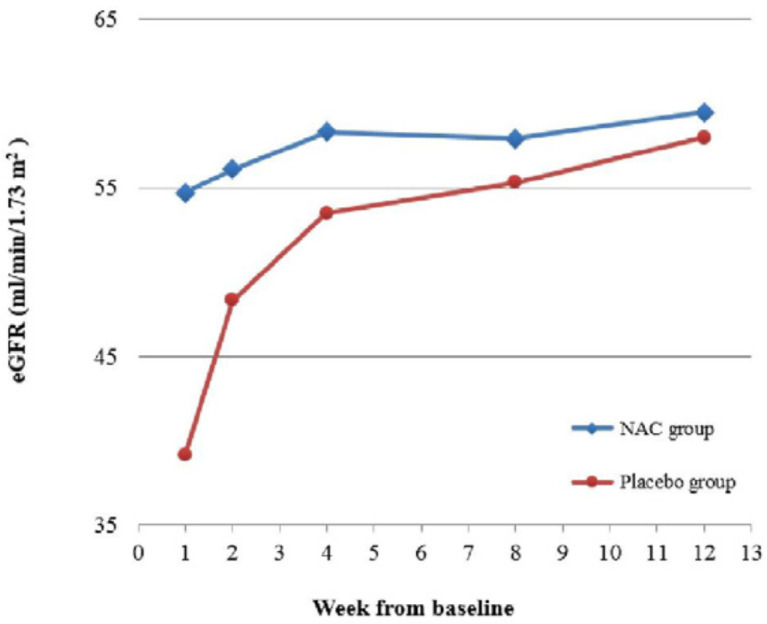
Estimated glomerular filtration rate during the 12-week follow-up

There was significant difference across the five time points in the eGFR, but more importantly, there was a significant interaction between time and group (*p* = 0.01). This indicates that although the eGFR in both groups improved over time, there was significant difference between the trends in each group (Table 2). The post-hoc analysis showed that the eGFR at week 1 was significantly higher in the NAC group (*p* = 0.01), but the difference between the two groups at the following time points were non-significant (all *p*-values ≥ 0.19). Considering the groups separately, the eGFR did not change significantly over time in the NAC group, indicating that it had already reached a stable condition by week 1. In the placebo group however, the eGFR increased significantly from week 1 to week 2 (*p* = 0.003), following which the change became nonsignificant. Figure 4 shows the change in eGFR for both groups, demonstrating considerably faster improvement of eGFR in the NAC group.

*Adverse effects*

Limited and mild adverse effects were reported during study intervention, including 2 (7.1%) nausea, 1 (3.6%) headache and 1 (3.6%) abdominal discomfort in the NAC group and 3 (13.6%) nausea, 2 (9.1%) flatulence and 1 (4.5%) headache in the placebo group (*p *= 0.40).

*Other findings*

The length of hospital stay was 12 days (10-17) vs. 14 days (12-21), *p* = 0.10. At the end of week 12, there was no recipient mortality. Two cases of graft loss were reported, one in each group. Acute rejection was observed in four patients, one in the NAC group vs. three in the placebo group.

**Table 1:** Baseline characteristics of donors and recipients

	Study groups		*p* value	
	**NAC group ** (n=28)	**Placebo group** (n=22)	t-test	Chi square
Donor age, years, mean±SD	36.8±13.6	40.6±15.9	0.37	
Male donor, n (%)	19 (67.8%)	19 (86.4%)		0.13
Expanded criteria donor, n (%)	9 (32.1%)	8 (36.4%)		0.75
Donors’ terminal serum creatinine, mean±SD	1.2±0.3	1.1±0.3	0.4	
Recipient age, years, mean±SD	41.6±12.2	46.0±12.0	0.21	
Recipient gender, n (%) Male Female	20 (71.4%) 8 (28.6%)	15 (68.2%) 7 (31.8%)		0.80
BMI , (Kg/m^2 ^), mean±SD	23.9±4.0	25.4±5.0	0.26	
Time on dialysis before transplantation, years, mean±SD	1.2±0.87	1.5±0.94	0.15	
Recipient baseline serum creatinine, mg/dl, mean±SD	7.3±2.5	7.2±1.9	0.83	
Pre-transplant NGAL level, pg/mL, mean±SD	1369.4±94.3	1326.2±21.5	0.91	
Cold ischemia time, hours, mean±SD	2.7±1.4	2.5±1.3	0.70	

* BMI, body mass index; eGFR, estimated glomerular filtration rate; NAC, N-acetylcysteine; SD: standard deviation. Expanded criteria donor was considered as: donors older than 50 years; or donors aged 40-49 years with two additional risk factors (hypertension, cerebrovascular accidents, serum creatinine more than 1.5 mg/dl, and diabetes without albuminuria).

**Table 2:** Study outcomes

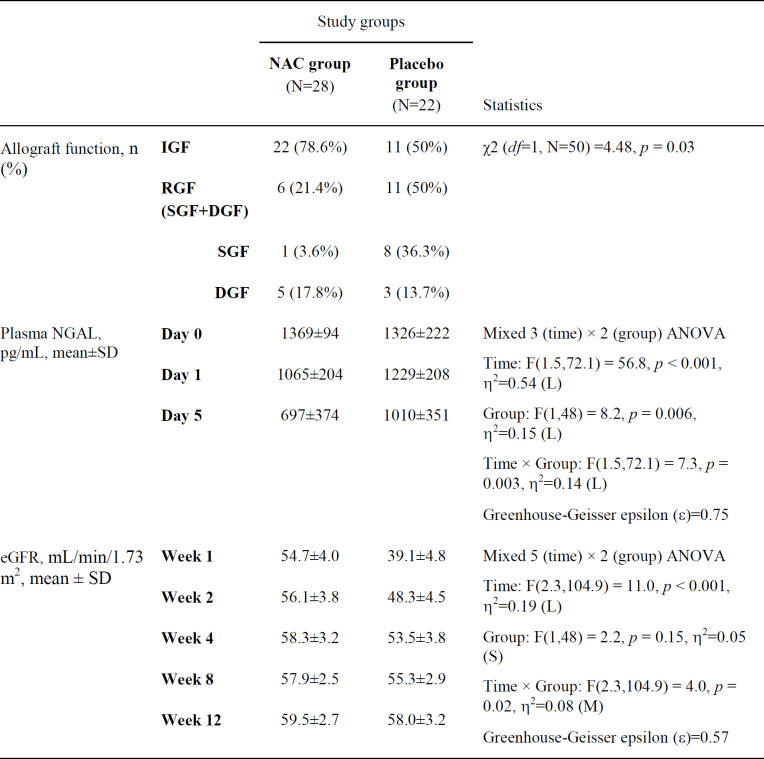

* IGF, immediate graft function; RGF, reduced graft function; SGF, slow graft function; DGF, delayed graft function; eGFR, estimated glomerular filtration rate; NAC, N-acetylcysteine. Effect sizes are indicated with the partial eta squared (^2^), with 0.01≤^2^≤0.059 indicating small (S), 0.06≤^2^≤0.139 indicating medium (M) and ^2^≥0.14 indicating large (L) effect size.

## Discussion

The results showed that the rate of RGF was significantly lower in the patients receiving NAC vs. placebo (21.4% vs. 50%). RGF is a common complication affecting renal grafts immediately post-transplantation and the clinical course of the patients with RGF is not as smooth as those recipients who have IGF. (5) Studies have used various time points and criteria in their definition of reduced graft function post-transplantation (24, 25). The conventional definition of DGF is the requirement for dialysis within the first week after renal transplantation. (26) In a study by Rodrigo et al DGF and SGF were defined as a CRR ≤30% with or without dialysis, respectively, on day 2 post-transplantation (25). Boom et al defined DGF when the serum creatinine remained unchanged, increased or decreased <10% per day on three consecutive days in the first week post-transplantation (24). Taking all this into consideration, the definition we used for RGF, which included DGF and SGF (CRR at day 7 post-transplantation of ≤70% with or without dialysis, respectively), incorporated all these definitions. Considering the rates of DGF and SGF separately, the former was not statistically different between the NAC and placebo groups (17.8% vs. 13.7%, respectively), however, the latter was substantially lower in the NAC group (3.6% vs. 36.3%). Overall, as stated above, the rate of RGF (DGF+SGF) was considerably lower in the NAC group. One of the possible mechanisms of reduced graft function is the ischemic damage to the graft before and during harvesting and is further aggravated by the reperfusion phase (27). The results from this study corroborate the findings by other studies that NAC has promising effects in reducing tubular injury and improving graft function. (12) The mechanism by which NAC improves the graft function primarily involves the scavenging of oxygen-free radicals, either directly or by increasing intracellular concentrations of glutathione, and vasodilation by increasing nitric oxide. The reported adverse effects were mild and transient and not statistically different between the two groups. Other trials of NAC also found no concern about its adverse effects (13, 28). The measurement of p-NGAL levels showed that the patients in the NAC group had significantly greater reduction of p-NGAL by both days 1 and 5 post-transplantation than those in the placebo group. The analysis showed that the patients in the placebo group had only 7.3% reduction in the p-NGAL levels by day 1 and 23.8% reduction by day 5. However, the patients receiving NAC achieved considerably faster reduction by day 1 (22.2%) and their p-NGAL reduced to almost half by day 5 (49.1%). The results thus showed that NAC can reduce tubular injury immediately after transplantation, evidenced by significantly lower levels of p-NGAL in the NAC group. A notable finding from comparing p-NGAL levels on day 1 and rate of IGF between the two groups was that p-NGAL may be considered as an early marker for immediate graft function. In the NAC group where the rate of IGF was considerably higher, the p-NGAL level dropped rapidly on day 1. In contrast, the placebo group, where the rate of IGF was lower, had modest drop in p-NGAL level on day 1. Several studies found that the p-NGAL measured within 24 h after transplantation correlates with the rate of IGF. Thus, the findings from this trial support other studies in that p-NGAL is an early and more specific marker for tubular injury and graft function (21). A near steady-state eGFR level was reached by week 1 in the NAC group, followed by a stable trend to week 12; however, the improvement of eGFR was significantly slower in the placebo group and a near steady-state was only achieved by week 4. In addition, the eGFR levels were always higher in the NAC than the placebo group. Comparing the trend of eGFR between the two groups showed that NAC led to faster and more sustained graft recovery during the first 12 weeks post-transplantation. Given NAC is safe and well-tolerated, higher dose and duration of its administration may be considered. A study by Sahraei et al on short-term administration of NAC (three doses of 600 mg NAC) did not show significant improvement in the graft function in renal transplant recipients. (28) On the other hand, the findings from the current study, also by Danilovic et al showed that 600 mg NAC consumed twice daily in the first week post-transplantation significantly improved tubular injury and graft function (12). In our study, the reduction of p-NGAL and rise of eGFR measured during the first week were significantly faster in the NAC vs. placebo group. However, from week 2, the difference between the two groups in the eGFR was nonsignificant. The notable efficacy of NAC in the first week and the plateaued effects thereafter opens an interesting debate on longer duration of NAC administration. In addition, since NAC undergoes extensive first-pass hepatic metabolism, its oral bioavailability is relatively poor (29). Therefore, future studies may also assess the effect of higher doses of oral NAC, such as 1200 mg which is commonly used in the prevention of contrast-induced nephropathy (29). The study had several limitations. First, it was a single center study. Second, the sample size was relatively small due to limited resources, however, statistically significant results were obtained for both primary and secondary outcomes, which shows that the results would be applicable to larger population. Third, preemptive renal transplantation and the patients receiving induction polyclonal antibody were excluded from study entrance in order to recruit a homogeneous population. The former patients have generally favorable graft outcomes than those undergoing maintenance hemodialysis before transplantation. The latter group was excluded to standardize the immunosuppressive regimen among study participants. Fourth, oxidative stress biomarkers were not assessed in the study. Nevertheless, there is a large body of evidence on the antioxidant and vasodilatory effects of NAC (10, 12, 30). Fifth, the follow-up was limited to 12 weeks, hence long-term graft survival was not assessed. Sixth, DGF is a multifactorial process in which several parameters can causes it such as many cytokines, chemokines, neutrophil activation, oxidant injury, etc. Between them, oxidant injury has a significant and comprehensive role (31, 32). Hence, we employed this indicator to evaluate the DGF. But the role of other factors should not be forgotten and they should be assessed in future researchers. In conclusion, the findings from this study showed that NAC has promising potential in reducing tubular injury and improving graft function. This was evidenced by significant reduction in the rate of RGF and levels of p-NGAL and increase in early-phase eGFR in the NAC group.
